# Revascularization Outcome Prediction for A Direct Aspiration-First Pass Technique (ADAPT) from Pre-Treatment Imaging and Machine Learning

**DOI:** 10.3390/brainsci11101321

**Published:** 2021-10-05

**Authors:** Tatsat R. Patel, Muhammad Waqas, Seyyed M. M. J. Sarayi, Zeguang Ren, Cesario V. Borlongan, Rimal Dossani, Elad I. Levy, Adnan H. Siddiqui, Kenneth V. Snyder, Jason M. Davies, Maxim Mokin, Vincent M. Tutino

**Affiliations:** 1 Canon Stroke and Vascular Research Center, University at Buffalo, Buffalo, NY 14203, USA; tatsatra@buffalo.edu (T.R.P.); mwaqas@ubns.com (M.W.); smousavi@buffalo.edu (S.M.M.J.S.); rdossani@ubns.com (R.D.); elevy@ubns.com (E.I.L.); asiddiqui@ubns.com (A.H.S.); ksnyder@ubns.com (K.V.S.); jdavies@ubns.com (J.M.D.); 2 Department of Mechanical and Aerospace Engineering, University at Buffalo, Buffalo, NY 14228, USA; 3 Department of Neurosurgery, University at Buffalo, Buffalo, NY 14203, USA; 4 Department of Neurosurgery and Brain Repair, University of South Florida, Tampa, FL 33613, USA; zren@usf.edu (Z.R.); cborlong@usf.edu (C.V.B.); mokin@usf.edu (M.M.); 5 Department of Pathology and Anatomical Sciences, University at Buffalo, Buffalo, NY 14203, USA; 6 Department of Biomedical Engineering, University at Buffalo, Buffalo, NY 14228, USA

**Keywords:** acute ischemic stroke, first pass effect, ADAPT, machine learning

## Abstract

A direct aspiration-first pass technique (ADAPT) has recently gained popularity for the treatment of large vessel ischemic stroke. Here, we sought to create a machine learning-based model that uses pre-treatment imaging metrics to predict successful outcomes for ADAPT in middle cerebral artery (MCA) stroke cases. In 119 MCA strokes treated by ADAPT, we calculated four imaging parameters—clot length, perviousness, distance from the internal carotid artery (ICA) and angle of interaction (AOI) between clot/catheter. We determined treatment success by first pass effect (FPE), and performed univariate analyses. We further built and validated multivariate machine learning models in a random train-test split (75%:25%) of our data. To test model stability, we repeated the machine learning procedure over 100 randomizations, and reported the average performances. Our results show that perviousness (*p* = 0.002) and AOI (*p* = 0.031) were significantly higher and clot length (*p* = 0.007) was significantly lower in ADAPT cases with FPE. A logistic regression model achieved the highest accuracy (74.2%) in the testing cohort, with an AUC = 0.769. The models had similar performance over the 100 train-test randomizations (average testing AUC = 0.768 ± 0.026). This study provides feasibility of multivariate imaging-based predictors for stroke treatment outcome. Such models may help operators select the most adequate thrombectomy approach.

## 1. Introduction

A direct aspiration-first pass technique (ADAPT) using large-bore catheters has emerged as a thrombectomy strategy that can rapidly reperfuse the afflicted vessels after acute ischemic stroke (AIS). Several studies and clinical trials have demonstrated that the ADAPT alone was successful in achieving a final revascularization modified thrombolysis in cerebral infarction (mTICI) 2b to 3 at a rate of 78%, with one or more attempts required to achieve successful reperfusion [1,2,3]. However, data show that the first pass effect (FPE, a modified treatment in cerebral ischemia-mTICI score of 2c and 3 after first pass) only occurs in 27% of ADAPT cases [2]. This is significant, as FPE has been associated with improved outcomes and lower peri-procedural complication rates [4].

Predicting which cases would most benefit from ADAPT, i.e., where ADAPT achieves FPE, would be highly valuable for selecting the proper thrombectomy approach. Several retrospective studies have reported single clinical and image-based parameters that correlate with successful aspiration-alone therapy [3,5,6,7]. However, many reports have conflicting findings, with no independent validation to support predictive claims. In this study, we test the feasibility of using combinations of measurable, pre-treatment imaging characteristics to predict FPE for ADAPT thrombectomies. To do this, we implemented a multivariate machine learning strategy with hold-out testing and model stability analyses.

## 2. Methods

### 2.1. Patient Population

This study was approved by the local Institutional Review Boards at the University at Buffalo in Buffalo, NY, USA (study number 030-403427) and the University of South Florida in Tampa, FL, USA (study number Pro00041063). Consecutive patients with acute ischemic stroke in the middle cerebral artery (M1) that were treated with ADAPT between July 2018 and June 2020 were included in this study. We retrospectively collected the patient images, both intra-procedural 2D digital subtraction angiogram (DSA) and CT imaging (nCCT and CTA), and patient medical data from consecutive cases. Cases were excluded if (1) ADAPT was not used as first pass technique, (2) the occlusion was not originating from the M1 segment of the MCA, (3) intra-procedural 2D DSA was not available, (4) CT imaging was not available, (5) the available CT imaging had significant artifacts, poor contrast opacification (i.e., no opacification detected by expert neuroradiologist) or poor definition of the clot (i.e., no hyperdensity sign on nCCT or missing collaterals, resulting in ambiguous definition of clot, especially clot length) and (6) patient or treatment information was missing.

### 2.2. CT Imaging

CT imaging was performed on an Aquilion ONE scanner (Canon Medical Systems Corporation, Otawara, Japan). The tube voltage and tube current were set to 135 kVp and 370–600 mA for nCCT, respectively, and 120 kVp and 150–205 mA for CTA, respectively. The reconstructed voxel sizes for the nCCT and CTA were set to 0.5 × 0.5 × 0.5 mm^3^ and 0.5 × 0.5 × 0.5 mm^3^, respectively. For CTA imaging, a dosage of 80 mL of Omnipaque 350 contrast was given at a rate of 5mL/s during CTA image acquisition.

### 2.3. ADAPT Procedure and Outcome Determination

Both centers employed a similar ADAPT treatment approach for the first MT pass [8,9]. In brief, a long sheath was placed into the distal cervical internal carotid artery. Following that, a large bore 0.068–0.072″ aspiration catheter (6 F Sofia (MicroVention/Terumo, Aliso Veijo, CA, USA), Jet 7 (Penumbra Inc., Alameda, CA, USA), React-71 (Stryker, Kalamazoo, MI, USA)) was advanced over the 0.025–0.035″ microcatheter and 0.014–0.016″ microwire (J-shaped Synchro2 (Stryker, Kalamazoo, MI, USA)) close to the proximal aspect of the clot. Aspiration was then performed via connection to a continuous aspiration pump. In cases where aspiration alone was not successful, stent retriever thrombectomy was performed next, in conjunction with continuous aspiration via the large-bore aspiration catheter.

Treatment success for our study was assessed on DSA immediately after the first pass ADAPT procedure using the mTICI score, based on the degree of reperfusion in the territory downstream of the occlusion: mTICI 2b (50–89% reperfusion), mTICI 2c (90–99% reperfusion) or mTICI 3 (100% reperfusion) [10]. First pass effect (FPE) was defined as having achieved mTICI scores of 2c or 3 at the end of the first use of MT [4].

### 2.4. Image Analysis

**Figure 1:**
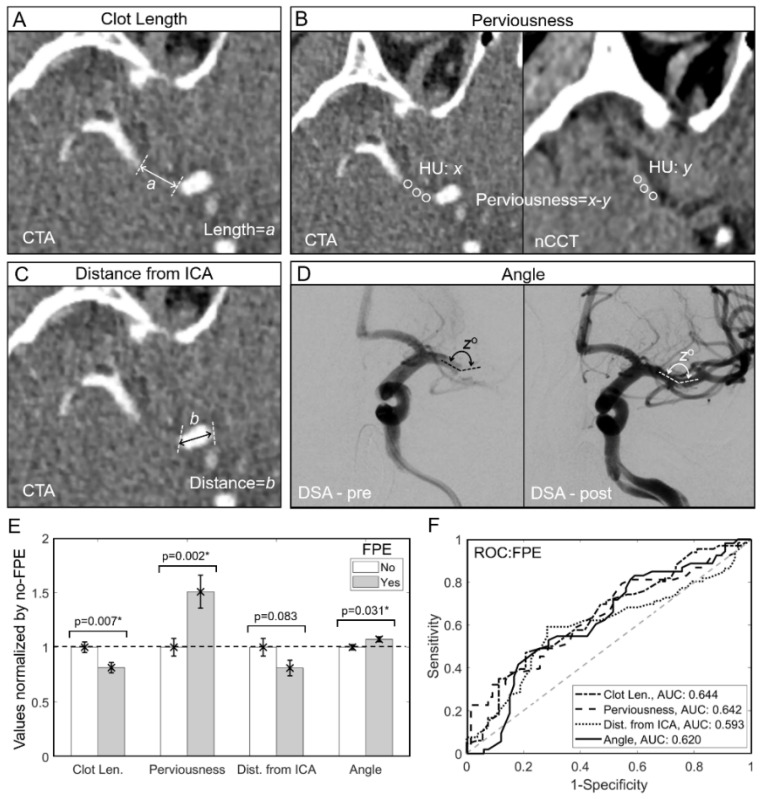
Clot image measurements and univariate analysis. (**A**) The coronal view of CTA, is used to measure clot length (*a*) using the largest extension of the filling defect on CTA. (**B**) Non-contrast computed tomography (nCCT) and computed tomography angiography (CTA) are co-registered, and 2–3 regions of interest along the clot area are used to calculate average density in Hounsfield units (HU) for each image. Perviousness is then calculated as the difference in HU values between CTA and nCCT (*x*-*y*). (**C**) To measure the distance from ICA terminus to the proximal end of the clot (*b*), a cumulative measured axial, coronal and sagittal CTA view was performed (not shown) to give adequate representation of distance on the axial CTA image alone. (**D**) On 2D digital subtraction angiography, the angle of interaction is measured as the angle between the aspiration catheter and the clot (dotted lines) from pre-thrombectomy working projections (*z*). This was then checked against final post-thrombectomy images. (**E**) A bar graph (error bars = standard error) of parameters normalized to the average value of the no-FPE group. Clot length, perviousness and angle of interaction were statistically different between the group that achieved FPE and the group that did not. (**F**) For each parameter, we performed ROC analysis to assess its ability in predicting FPE. Clot length and perviousness had the best AUCs, followed by angle of interaction and distance from the ICA. Abbreviations: AUC = area under the curve, CTA = computed tomography angiography, Dist. = distance, DSA = digital subtraction angiography, FPE = first pass effect, HU = Hounsfield units, ICA = internal carotid artery, Len. = length, nCCT = non-contrast computed tomography, ROC = receiver operating characteristic. * denotes statistical significance, *p* < 0.05.

Image analysis was performed to calculate perviousness, clot length, angle of interaction (AOI) and the distance of clot from the ICA terminus. As shown in Figure 1A–D [6,9,11], they were computed as follows:

We calculated clot length as previously described (Figure 1A) [9,12]. To define the clot region, we identified the largest extension of the filling defect on CTA. This was then manually measured on the optimal coronal CTA view (i.e., the plane that included the majority of the clot region). In cases of clot extending into multiple MCA bifurcation or trifurcation branches, the longest clot length was used for calculations. Cases where there were no hyperdensity signs on nCCT or an absence of collaterals on CTA were excluded because the clot length was ambiguous and could not be computed.

We calculated clot perviousness as described elsewhere (Figure 1B). Ref. [13,14] For clots that were hyperdense on nCCT, the CTA and nCCT images were placed side-by-side to identify the clot region. For clots that were not hyperdense on nCCT, the CTA and the corresponding nCCT images were co-registered using the open source software, 3D Slicer (https://www.slicer.org/, accessed on 15 April 2021), which implemented the BRAINSFit algorithm for mutual-information rigid registrations of whole-brain 3D images [15]. Next, 2–3 regions of interest in the clot region on both nCCT and CTA were chosen and their Hounsfield units (HU) were averaged. Perviousness was calculated as the difference between average HU from CTA and nCCT.

The distance of the proximal end of the clot from the ICA terminus was calculated on CTA images (Figure 1C). We assessed the standard CTA image projections (i.e., axial, coronal and sagittal views) in order to identify the optimal projection on which the distance between the clot and the end of the ICA was captured. This distance on CTA was then manually measured.

We measured the AOI (angle of interaction) between the clot and the aspiration catheter, as described elsewhere (Figure 1D) [6]. In brief, the AOI is calculated as the angle between the major axes of the aspiration catheter and the clot using 2D DSA. Optimal, in-plane projections are used to identify the axes where the catheter and the clot lie.

### 2.5. Univariate Statistical Analysis

In our dataset, the patients were divided into two groups based on the achievement of FPE after ADAPT. First, we performed univariate statistical analyses to assess differences in patient clinical parameters and clot-related image features (clot length, perviousness, distance from the ICA and interaction angle) between patients with first pass effect and those without first pass effect. For categorical variables, we performed the Fisher’s exact test to test for significance. For continuous variables, Sharpiro–Wilk (SW) tests were first performed to test each continuous parameter for normality. Then, in order to test significance, we performed a Student’s *t*-test (for parameters that were normally distributed) or a Mann–Whitney U-test (for parameters that were not normally distributed). Pearson correlation analysis was performed and the correlation coefficient and *p*-value reported to test the collinearity between the imaging parameters. DeLong’s test was performed to compare ROCs. Results were reported as mean ± standard error (SE). A parameter was considered significant if *p* < 0.05. For each image-based feature, we also plotted the receiver operator characteristic (ROC) curve, and calculated the area under the ROC curve (AUC) in order to measure its performance in predicting FPE. All analyses except DeLong’s test were performed in MATLAB (v.2020a, MathWorks, Natick, MA, USA). DeLong’s test was performed in MedCalc Statistical Software (v.19.2.6, MedCalc Software Ltd., Ostend, Belgium).

### 2.6. Multivariate Machine Learning Analyses

In order to determine if a combination of imaging parameters could better predict first pass effect than each individual metric, we performed multivariate machine learning (ML) analyses. We first divided our dataset randomly into a training and testing cohort using a 75%:25% split (n = 89 and n = 30 cases, respectively), maintaining equal proportions of cases that did and did not achieve FPE. Then, prior to ML, the input data were standardized to a median of 0 and an IQR of 1 based on the training data, to facilitate faster and more accurate convergence of the models. In the training dataset we trained logistic regression (LR), linear discriminant analysis (LDA) and support vector machine (SVM—with a linear kernel) classifiers in the scikit-learn ML library in Python v3.7.4. To minimize overfitting, we implemented a 10-fold cross-validation (CV) in the training cohort for hyperparameter optimization in the chosen algorithms. The final model that used all four measured parameters was trained in the complete training dataset with the hyperparameters optimized using 10-fold CV. To estimate their performance, we calculated their training accuracy, sensitivity, specificity and AUC (with 95% CI from CV). The models were then tested in the hold-out testing cohort to determine their true performance, via calculation of their testing accuracy, sensitivity, specificity and AUC (with 95% CI from CV).

To assess the improvement in performance of multivariate models over the individual metrics themselves, we performed univariate ‘model training’ in the training dataset for these parameters. In brief, we performed ROC analysis for each of the 4 parameters on the training cohort and computed the optimal cutoff for FPE prediction using the Youden’s J index. To compare to model performance, we recorded the univariate training ROCs and training accuracy, sensitivity and specificity. We also assessed individual metric predictions in the testing cohort, and reported the testing accuracy, sensitivity and specificity there as well.

### 2.7. Model Stability Testing

As these results may be dependent on the specific randomization of training and testing, we tested the stability of these models by repeating the multivariate ML procedure across 100 randomizations of training/testing. For this analysis, the average training and testing accuracy, sensitivity, specificity and AUC (with 95% CI) across the 100 randomizations were calculated and compared to the performance of the models in the single randomization.

## 3. Results

### 3.1. Patient Population

A total of 119 patients were included in the study. Overall patient characteristics of the dataset have been reported in Table 1. Of the 119 cases (71 from Buffalo, NY, and 48 from Tampa, FL) included in the study, 53 patients (44.5%) achieved FPE (i.e., mTICI of 2c and 3 after first pass). Seventy patients (58.8%) were female and the mean age of the cohort was 72.8 ± 1.4 years. There was no significant difference between patients with and without FPE in terms of gender proportions, mean age, comorbidities, IV tissue plasminogen alteplase (TPA) administration, laterality proportions and LVO location proportions (see Table 1).

### 3.2. Shorter Length, Higher Perviousness and Larger AOI Are Associated with FPE

Figure 1E shows that cases that achieved FPE had significantly shorter clots (9.79 ± 0.57 mm vs. 12.05 ± 0.58 mm, SW-test, *p* = 0.021, *t*-test *p* = 0.007), higher perviousness (37.60 ± 3.74 HU vs. 24.92 ± 2.02 HU, SW-test, *p* < 0.001, *t*-test, *p* = 0.002) and larger AOI (149.91 ± 3.11° vs. 139.77 ± 3.32°, SW-test, *p* < 0.001, *t*-test, *p* = 0.031) compared to those that did not achieve FPE. Cases of FPE also tended to have a shorter distance from the ICA terminus (11.64 ± 1.01 mm vs. 14.37 ± 1.28 mm, SW-test, *p* = 0.004, *t*-test, *p* = 0.083), albeit this difference was not statistically significant. Figure 1F demonstrated the ROC curves for each of these parameters across the entire dataset. Individually, no parameter performed above an AUC of 0.65. Clot length and perviousness had the highest predictive ability for FPE with AUC = 0.644 and AUC = 0.642, respectively. AOI and distance from the ICA had AUC = 0.620 and AUC = 0.593, respectively. Before multivariate model training, we performed univariate collinearity testing, and found no linear correlations between any variable pairs (see Supplemental Figure S1).

### 3.3. Machine Learning Models Predict FPE with Good Accuracy

**Figure 2:**
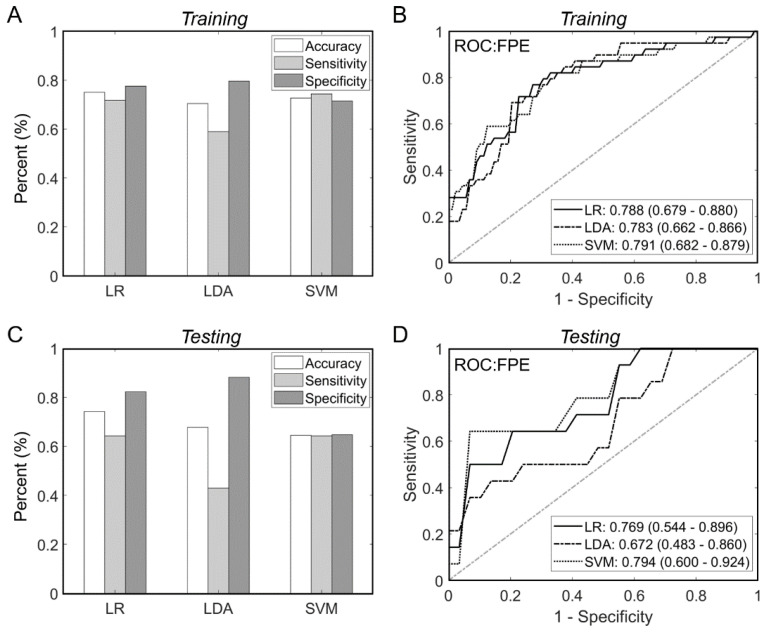
Multivariate machine learning analysis. (**A**) We trained three ML models (LR, LDA and SVM) to use normalized values of all four parameters to predict FPE. In training, the models performed with an accuracy of ranging from 70%–75%. (**B**) ROC analysis in the training dataset demonstrated that the models had an AUC ranging from 0.78–0.79. (**C**) In testing, the models had an accuracy ranging from 65%–74%, with the LR modeling performing the best. (**D**) The models AUC in the testing dataset ranged from 0.67–0.79. Abbreviations: AUC = area under the curve, FPE = first pass effect, LDA = linear discriminant analysis, LR = logistic regression, ML = machine learning, ROC = receiver operating characteristic, SVM = support vector machines.

To test if multivariate ML models perform better than univariate FPE predictions, we trained and tested LR, LDA and SVM algorithms with the four image-based parameters to predict FPE. The optimal hyperparameters were computed using the 10-fold internal CV on the training dataset and presented in Supplemental Table S1. As shown in Figure 2A, the LR, LDA and SVM models achieved training accuracies of 75.0%, 70.6%, and 72.7%, respectively. In general, the models had higher specificity (LR: 77.6%, LDA: 79.6%, SVM: 71.4%) than sensitivity (LR: 71.8%, LDA: 59.0%, SVM: 74.4%). As shown in Figure 2B, the AUC analysis for the trained models yielded ROCs of 0.788 (95% confidence interval–CI: 0.679–0.880), 0.783 (95% CI: 0.679–0.880) and 0.791 (95% CI: 0.662–0.866) for the LR, LDA and SVM models.

In the hold-out testing cohort, the algorithms had similar accuracies and AUCs. The most accurate model was LR (74.2%), followed by LDA (67.7%) and SVM (64.5%), as shown in Figure 2C. Our ROC analysis demonstrated AUCs of 0.769 (95% CI: 0.544–0.896), 0.672 (95% CI: 0.483–0.860) and 0.794 (95% CI: 0.600–0.924) for the LR, LDA and SVM models, respectively (Figure 2D). (See Supplemental Table S2 for equations of the models). Interestingly, the multivariate LR model outperformed any of the 4 univariate models derived based on their individual optimal cutoffs on the training dataset (refer to Supplemental Figure S2).

### 3.4. The Logistic Regression Model Is Stable over 100 Training/Testing Randomizations

**Figure 3:**
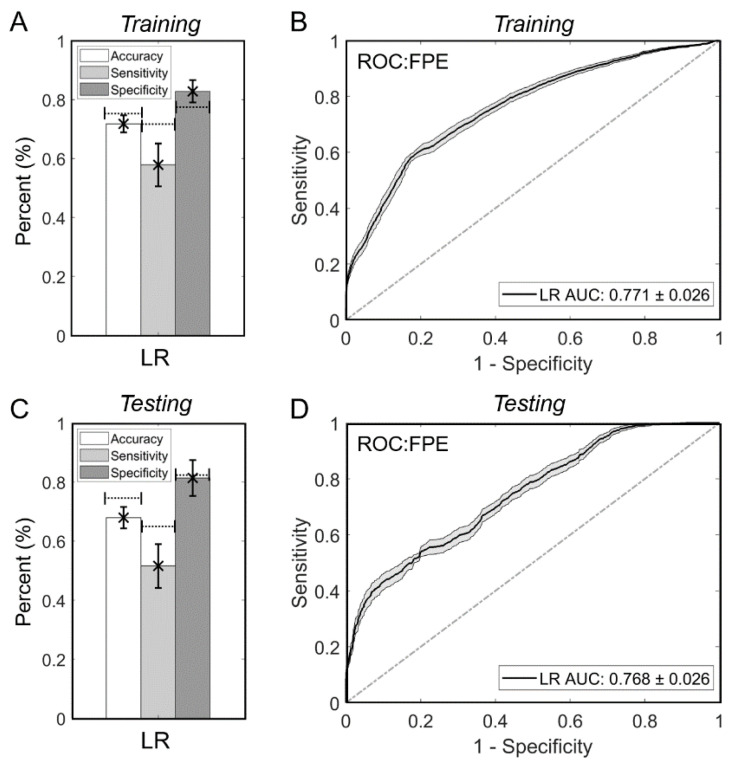
LR model stability across 100 randomizations of training/testing. (**A**) Across 100 different randomizations of the selection of the training dataset, the LR model had an accuracy, sensitivity and specificity of 0.717 ± 0.029, 0.578 ± 0.073 and 0.828 ± 0.038, respectively. This was similar to the performance in the initial randomization, illustrated by the dashed lines. (**B**) The AUC measured in the first randomization also fell within the standard error range across the 100 training AUCs, 0.771 ± 0.026. (**C**) In the 100 different testing dataset randomizations the LR model had an average testing accuracy, sensitivity and specificity of 0.679 ± 0.036, 0.518 ± 0.074 and 0.814 ± 0.061, respectively. This was again similar to the testing results in the initial randomization, illustrated by the dashed lines. (**D**) The testing AUC measured in the first randomization also fell within the SE range across the 100 testing AUCs, 0.768 ± 0.026. Abbreviations: AUC = area under the curve, FPE = first pass effect, LR = logistic regression, ROC = receiver operating characteristic.

To test the stability of the LR model, we repeated the ML workflow for LR over 100 randomizations of the training/testing split. For each randomization the optimal hyperparameters were computed with the 10-fold internal CV on the training dataset. As shown in Figure 3A, the LR model achieved an average training accuracy, sensitivity and specificity of 71.7 ± 2.9%, 57.8 ± 7.3% and 82.8 ± 3.8%, respectively, over the 100 randomizations. Furthermore, its average training AUC was 0.771 ± 0.026 (Figure 3B). Across the 100 random testing cohorts, the LR model performed with average testing accuracy, sensitivity and specificity of 67.9 ± 3.6%, 51.8 ± 7.4% and 81.4 ± 6.1%, respectively (Figure 3C). Its average testing AUC was 0.768 ± 0.026 (Figure 3D). Similar trends of results were observed with SVM and LDA classifiers trained and tested on the same 100 random train-test splits (see Supplemental Figure S3). As shown by the dotted lines in Figure 3A,C, the single-randomization trained LR model had similar performance to that of the 100 randomizations, although its accuracy and sensitivity tended to be higher. Nevertheless, the testing and training AUC values of the model (from the single randomization), were well within the AUC range calculated from the 100 randomizations.

### 3.5. Interpreting the Logistic Regression Model

The logistic regression model allows for direct calculation of the odds ratio (OR) for each parameter based on the trained coefficients (refer Supplemental Table S2). Table 2 shows the odds ratio and the predicted percent change in positive outcomes in our dataset (which had a positive outcome rate of 44.5%) with an increase in each parameter by 1 IQR [16]. For example, a 7 mm (1 IQR) increase in clot length, will decrease the rate of positive outcomes in the dataset by 25.87%. Based on these findings, the most important parameters were (in decreasing order), clot length, AOI, distance of the clot from the ICA terminus, and perviousness. Based on the order of importance (from %change in outcome), these metrics can be ranked into a simple decision tree (Supplemental Figure S4). The output of each branch of the tree in Supplemental Figure S4 shows the predicted probability of outcome for a ± 1 IQR deviation of each metric.

## 4. Discussion

In this study, we created a regression model that predicted FPE for ADAPT cases in the MCA with an accuracy of 74.2% and an AUC of 0.77 stable across iterative ML analyses. It is likely that this model worked well because the features it relied on, themselves, all reflect different facets of successful or unsuccessful first pass attempts. Physical properties of the clot may reflect the ease to which it can be rapidly removed from the vessel by aspiration. In our data, FPE was significantly associated with smaller clot length (*p* = 0.007). Guzzardi et al. also found that shorter clots were associated with better clinical outcomes in ADAPT [1]. This may be because longer surface area clots have larger friction and adhesion forces against the vessel, making them harder to retrieve and yielding worse thrombectomy outcomes [17]. Similarly, higher perviousness was also significantly associated with FPE (*p* = 0.002). Perviousness has been shown to be associated with greater proportions of fibrin mesh and has been demonstrated to be related to the degree of recanalization with IV-tPA administration alone [13,18]. It has been hypothesized that higher perviousness allows for more tPA to pass through the clot, which could result in faster thrombus breakdown and better recanalization [19]. Similarly, for ADAPT cases, greater penetration of the blood into the clot material in those that are more pervious could enable the suction of distal blood, which would benefit the removal of the clot.

The position of the clot in the cerebral vasculature may also influence the ease with which it can be retrieved. Based on our data, the AOI between the clot and the aspiration catheter was significantly greater in cases that achieved FPE (*p* = 0.031). Indeed, Bernava et al. originally demonstrated that the final recanalization outcome and number of passes until revascularization (rather than FPE) were significantly associated with greater angle of interaction to the clot [6]. This is likely because, as the AOI approaches 180° (where the aspiration catheter is coaxial to the clot), the entire aspiration force is applied to the clot, which would lead to easier aspiration and more effective reperfusion. Additionally, we also found that FPE occurred more often in cases where the clot was closer to the ICA terminus, albeit the difference was not statistically significant (*p* = 0.083). While others have also reported no difference in FPE across different clot locations, one could still speculate that FEP could be related to shorter distance from the ICA because 1) distal clots may be more susceptible to breakdown and/or loss from the catheter tip when it is being withdrawn and 2) due to decreased contrast opacification, it may be difficult to visualize the location of distal clots, thus resulting in suboptimal contact between the aspiration catheter and the clot [5,20].

Recent studies have identified other predictors of functional and reperfusion outcomes after mechanical thrombectomy [21,22,23]. These primarily include demographic features, medical history and preliminary stroke characteristics. For example, Alexandre et al., in a retrospective, multi-center, observational study of 191 patients with posterior circulation occlusion, found lower baseline NIHSS and higher ASPECTS (Alberta Stroke Program Early CT Score) to be predictors of better functional outcome, and the use of larger bore catheters as a positive predictor of successful reperfusion [21]. Focusing on FPE, Velagupadi et al. investigated a cohort of 220 patients who underwent MT, and used demographic characteristics, medical history and stroke characteristics for machine learning analysis of first pass reperfusion prediction [22]. Testing various ML algorithms—SVM, Random Forest, Naïve Bayes, LR, and XGBoost, they reported an accuracy ranging from 60%–67% in predicting FPE. Di Maria et al. also aimed to predict FPE using medical record data [23]. In a cohort of 1832 patients, they found multiple features, including age, blood pressure and location, were independent predictors of first pass reperfusion. These studies, although they did not attempt to identify image-based FPE predictors, provide evidence that patient data (like demographics and medical history) may also help in predicting FPE. Future studies investigate how the addition of these features to our models could increase performance, as warranted.

The overarching goal of this study was to test the feasibility using ML based solely on imaging metrics to create predictive models of FPE for ADAPT cases. In comparing the algorithms’ performance with univariate analyses of the individual parameters, it is clear that combining the parameters led to better FPE prediction than could be achieved by any individual parameter alone. While most were significantly different between cases that did and did not achieve FPE, their AUCs across the entire dataset were generally poor, ranging from 0.59–0.64. Their AUCs were equally poor in the training dataset, where they fell to 0.51–0.56. Their low predictive ability, likely due to inter-sample variation in the metrics, may be one reason for the non-significant and inconsistent results that have been reported in the literature [9,11,24,25]. Taking a different approach than other studies, we created classification algorithms that use discrete feature values from each case to predict FPE, rather than simply assessing average differences across the two populations. This ML strategy enabled us to create predictors that had substantially higher AUCs (as high as 0.79 (95% CI: 0.600–0.924) in the testing dataset). This shows that combinations of features, which may be individually inconsistent, can generate stable predictions and accommodate for potential inter-sample variability in the data. Such multivariate models are particularly powerful when the parameters used in their model building are not correlated (collinearity testing), as was the case in our dataset, where each parameter was both philosophically and statistically related to different aspects of treatment outcome, thereby allowing for better model performance [26].

Our study has several limitations. First, we only analyzed ADAPT, which could have introduced bias towards cases preferentially selected for aspiration, e.g., those with favorable anatomy. Choice of ADAPT over other strategies is dependent on other factors (including personal experience and device availability) that could hinder the use of predictive models like the ones developed here. Second, these findings are only applicable to patients with occlusions originating in the M1 segment of the MCA, since we excluded patients with occlusions at other locations. Third, there are limitations to each parameter we analyzed in this study. Clot length requires cases to have well-defined filling defects (see inclusion/exclusion criteria), which may have biased our population. The perviousness calculation may vary based on partial volume effects in the quantified regions, collateral quality and atherosclerotic lesions in the vessel wall. Distance from the ICA terminus was measured in this study on 2D CT planes. However, center-line analyses on 3D reconstructions would offer a more accurate way to measure this parameter. AOI is tedious to obtain from pre-treatment CT imaging, as it would require clot and proximal vessel segmentation. Fourth, we did not include device-related parameters in our ML models. For example, aspiration catheter bore diameter may increase model performance, as a recent study showed it to be independently associated with FPE [27]. Future studies that include MT device parameters along with imaging parameters are needed. Lastly, future validation studies in larger, multi-center cohorts are needed to establish the predictive ability of this model.

## 5. Conclusions

This preliminary study provided evidence that combinations of image-based parameters could predict FPE in ADAPT cases. Our data showed that these ML models predicted FPE better than individual metrics alone. In particular, a LR model showed a hold-out testing accuracy of and AUC of 0.742 and 0.769, respectively. Combinations of parameters may give more stable FPE predictions by accounting for inter-sample variability in the individual parameters. This preliminary study provides the foundation for development of a multi-variate imaging-based outcome predictor for ADAPT-treated large vessel occlusions. Following rigorous validations in large, multi-cohort studies, such models may help the operators select the most adequate MT approach for each individual patient.

## Figures and Tables

**Table 1:** Comparison of baseline characteristics of groups with and without FPE.

	FPE (n = 53)	No FPE (n = 66)	*p*-Value
**Demographic and Clinical Data**			
Male, n(%)	21 (39.6)	28 (42.4)	0.852
Female, n(%)	32 (60.4)	38 (57.6)	
Age, (mean ± SE)	75.7 ± 2.0	70.5 ± 1.9	0.076
Hypertension, n(%)	42 (79.3)	49 (74.2)	0.663
Diabetes mellitus, n(%)	14 (26.4)	18 (27.3)	1.000
Dyslipidemia, n(%)	24 (45.3)	32 (48.5)	0.854
Congestive heart failure, n(%)	6 (11.3)	10 (15.2)	0.599
Atrial fibrillation, n(%)	17 (32.1)	32 (48.5)	0.092
Current smoker, n(%)	7 (13.2)	7 (10.6)	0.777
Previous stroke, n(%)	9 (17.0)	17 (25.8)	0.273
**Treatment Details**			
IV-tPA, n(%)	33 (62.3)	30 (45.5)	0.065
**Stroke Presentation**			
Right-sided occlusion, n(%)	33 (62.3)	34 (51.5)	0.268
Left-sided occlusion, n(%)	20 (37.7)	32 (48.5)	
MCA M1 occlusion, n(%)	44 (83.0)	44 (66.7)	0.058
MCA M2 occlusion, n(%)	9 (17.0)	22 (33.3)	
Clot density-nCCT, HU (mean ± SE)	42.5 ± 1.5	41.2 ± 1.3	0.540
**Image-Based Parameters** (All parameters normally distributed based on SW test)			
Clot length, mm (mean ± SE)	9.79 ± 0.57	12.05 ± 0.58	0.007 ^†^
Clot perviousness, HU (mean ± SE)	37.60 ± 3.74	24.92 ± 2.02	0.002 ^†^
Distance from ICA, mm (mean ± SE)	11.64 ± 1.01	14.37 ± 1.28	0.083
Angle of interaction (mean ± SE)	149.1 ± 3.11	139.77 ± 3.32	0.031 ^†^

HU = Hounsfield units, ICA = internal carotid artery, ICA = internal carotid artery, IV = intravenous, n = number, nCCT = non-contrast computed tomography, MCA = middle cerebral artery, SE = standard error, tPA = tissue plasminogen activator. ^†^ Indicates significance (*p* < 0.05).

**Table 2:** Statistics from the trained coefficients of the LR model.

Parameter.	Odds Ratio	Median	IQR	*p_change_*
Clot Len.	0.2859 ± 0.0148	10 mm	7 mm	−25.87%
Perviousness	1.6356 ± 0.0383	27 HU	24 HU	12.15%
Dist. From ICA	0.3852 ± 0.0142	12 mm	12.8 mm	−20.92%
AOI	2.7292 ± 0.0382	150°	29°	24.06%

Odds ratio, training cohort median, training cohort inter-quartile range, and calculated %change in positive outcome rate with unit IQR increase in each independent variable. Abbreviations: AOI = angle of interaction, Dist. = distance, HU = Hounsfield units, ICA = internal carotid artery, IQR = interquartile range, Len. = length, LR = logistic regression.

## Data Availability

All data relevant to the study are present in the manuscript and the supplementary material.

## Supplementary Materials

The following are available online at https://www.mdpi.com/article/10.3390/brainsci11101321/s1, Supplemental Table S1: Range and Optimal Hyperparameters Computed from 10-Fold Internal Cross-Validation in Training, Supplemental Table S2: Equations of the Machine Learning Models, Supplemental Figure S1: Univariate Collinearity Statistics for the Four Independent Variables Used in Logistic Regression Training, Supplemental Figure S2: Univariate ‘Model’ Training and Testing Performance of Individual Parameters, Supplemental Figure S3: LDA (Red) and SVM (Blue) Model Stability Across 100 Randomizations of Training/Testing, Supplemental Figure S4: Inferred Decision Tree from Trained Logistic Regression Model Coefficients.
