# Supplementary material for: Revascularization Outcome Prediction for A Direct Aspiration-First Pass Technique (ADAPT) from Pre-Treatment Imaging and Machine Learning
Source: Brain Sci. 2021 Oct 5;11(10):1321. doi: 10.3390/brainsci11101321 (PMC8534082; doi:10.3390/brainsci11101321)
Supplement: Supplementary file 1 [file brainsci-11-01321-s001.zip › brainsci-1381243-supplementary.pdf]

Supplemental Information for: Revascularization Outcome Prediction for A Direct Aspiration-First Pass Technique (ADAPT) from Pre-Treatment Imaging and Machine Learning

Tatsat R. Patel [1,2], Muhammad Waqas [1,3], Seyyed M.M.J. Sarayi [1,2], Zeguang Ren [4], Cesario V. Borlongan [4], Rimal Dossani [1,3], Elad I. Levy [1,3], Adnan H. Siddiqui [1,3], Kenneth V. Snyder[1,3], Jason M. Davies [1,3], Maxim Mokin [4] and Vincent M. Tutino [1,2,3,5,6,*]

[1] Canon Stroke and Vascular Research Center, University at Buffalo, Buffalo, NY 14203, USA; tatsatra@buffalo.edu (T.R.P.); mwaqas@ubns.com (M.W.); smousavi@buffalo.edu (S.M.M.J.S.); rdossani@ubns.com (R.D.); elevy@ubns.com (E.I.L.); asiddiqui@ubns.com (A.H.S.); ksnyder@ubns.com (K.V.S.); jdavies@ubns.com (J.M.D.)

[2] Department of Mechanical and Aerospace Engineering, University at Buffalo, Buffalo, NY 14228, USA

[3] Department of Neurosurgery, University at Buffalo, Buffalo, NY 14203, USA

[4] Department of Neurosurgery and Brain Repair, University of South Florida, Tampa, FL 33613, USA; zren@usf.edu (Z.R.); cborlong@usf.edu (C.V.B.); mokin@usf.edu (M.M.)

[5] Department of Pathology and Anatomical Sciences, University at Buffalo, Buffalo, NY 14203, USA

[6] Department of Biomedical Engineering, University at Buffalo, Buffalo, NY 14228, USA

* Correspondance: vincentt@buffalo.edu; Tel./Fax: (716) 829-5400

***Corresponding Author:**
Vincent M. Tutino, PhD
E-mail: vincentt@buffalo.edu
Phone: (716) 829-5400
Fax: (716) 829-5400

Running Title: Machine Learning to Predict First Pass Effect

Supplemental Tables

Supplementary Table S1: Range and Optimal Hyperparameters Computed from 10-Fold Internal Cross-Validation in Training.

Model	Hyperparameters	Range	Optimized value
LR	*Penalty*	‘l1’, ‘l2’, ‘elasticnet’	‘l2’
	Regularization parameter ‘C’	$[10^{-3},10^{2}]$	10
	Tolerance	$[10^{-7},10^{4}]$	10^{-4}
	Solver	‘newton-cg’, ‘lbfgs’, ‘liblinear’, ‘sag’, ‘saga’	‘lbfgs’
LDA	*Solver*	’svd’, ‘eigen’, ‘lsqr’	‘lsqr’
	Tolerance	$[10^{-7},10^{4}]$	10^{-4}
SVM	*Regularization parameter ‘C’*	$[10^{-2},10^{1}]$	10^{-1}
	Kernel	‘linear’	‘linear’
	Tolerance	$[10^{-7},10^{4}]$	10^{-3}

Supplementary Table S2: Equations of the Machine Learning Models.*

Model	Equation
LR†	$ODDS = e^{(-1.252*length + 0.492*pervious + 1.004*AOI - 0.954*dICA - 0.152)}$ $P = \frac{ODDS}{1 + ODDS}$
LDA	$P = -0.837 * Length + 0.592 * Pervious + 0.889 * AOI - 0.534 * dICA - 0.211$
SVM	$P = -1.015 * Length + 0.197 * Pervious + 1.035 * AOI - 0.795 * dICA + 0.111$

* † $IF\ P \geq 0.5, predicted\ FPE,\ IF\ P < 0.5, predicted\ no\ FPE$. From calculation of ODDS, the probability of achieving FPE is then derived from the equation for P. We note that in calculating FPE probability, parameters must first be normalized to along a zero median and an IQR of 1, as described in the methods.

Supplemental Figures

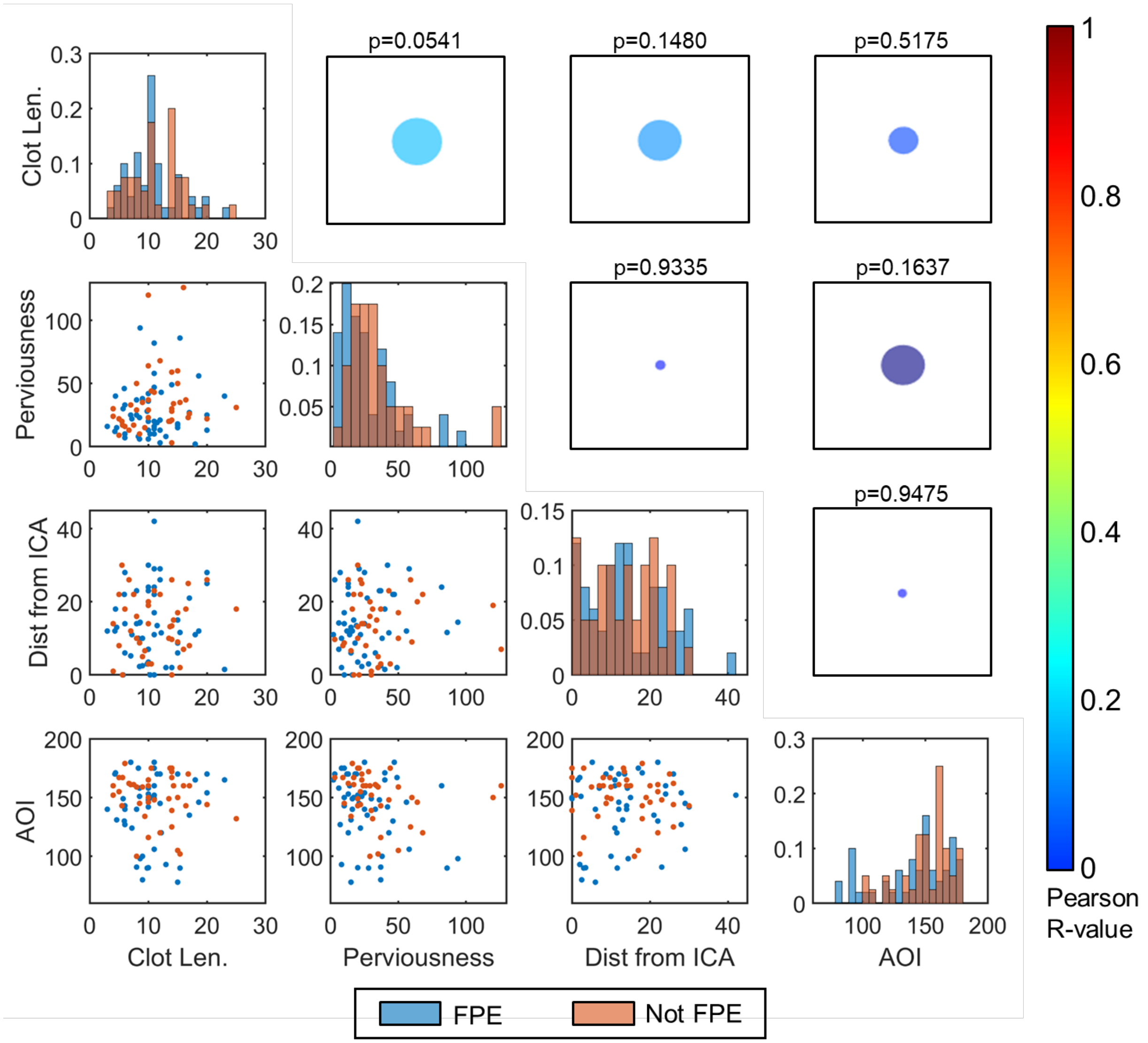

Supplementary Figure S1: Univariate Collinearity Statistics for the Four Independent Variables Used in Logistic Regression Training. The plots along the diagonal indicate the normalized distributions of the 4 variables segregated in terms of FPE and not FPE cases. The plots be below the diagonal indicate the scatter plots of each combination of 2 variables, again segregated by FPE and not FPE cases. The plots above the diagonal indicate the Pearson linear correlation coefficients based on their diameter and color (see color bar on the right), along with their p-values.

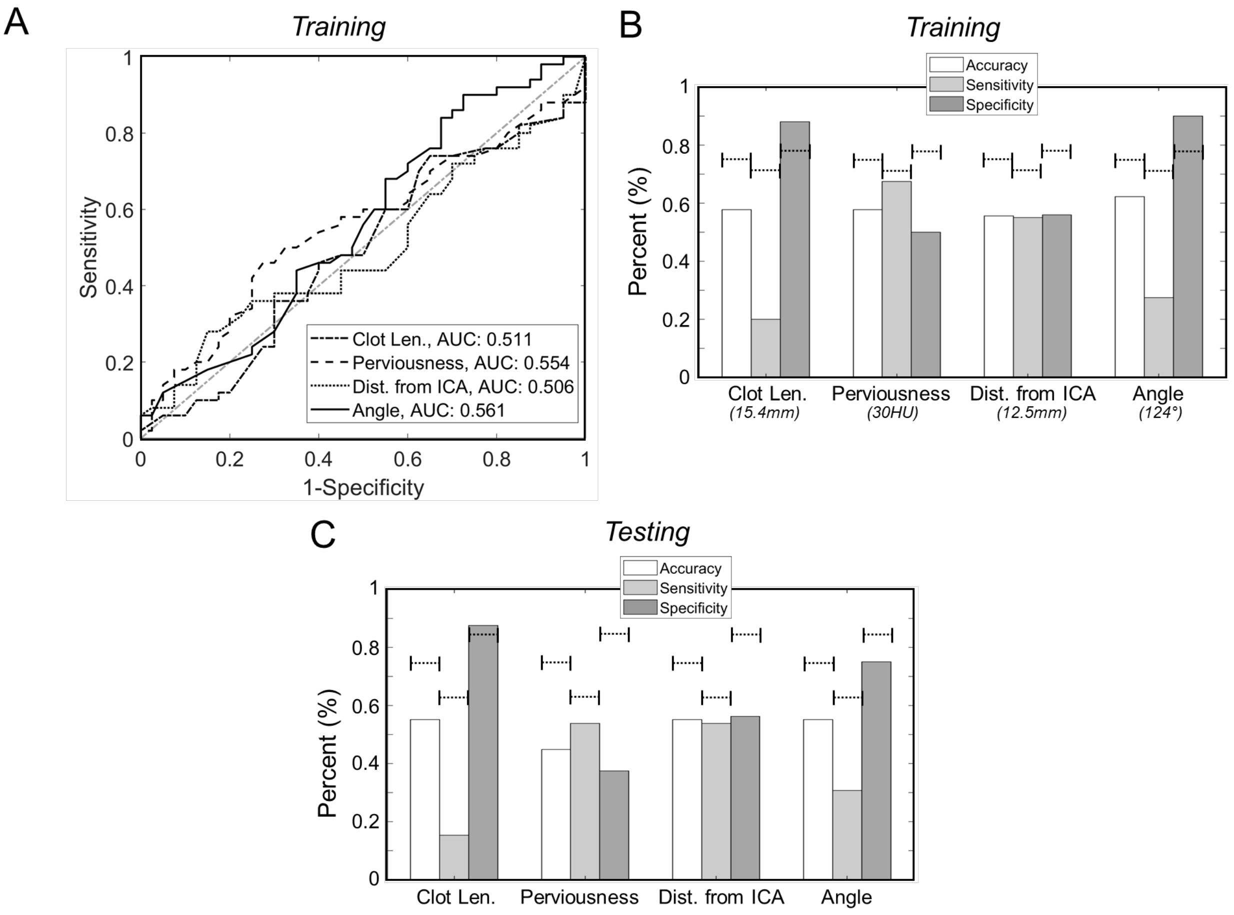

Supplementary Figure S2: Univariate 'Model' Training and Testing Performance of Individual Parameters. (A) ROCs and their corresponding AUCs in the training dataset. **(B)** Based on the optimal cut-offs (mentioned below the parameter) computed using Youden's index from the ROC analysis, the accuracy, specificity and sensitivity of all 4 parameters on the training dataset. The dashed lines indicate the performance of the LR model in training cohort. **(C)** Using the optimal cut-off, the accuracy, specificity and sensitivity of all 4 parameters on the testing dataset. The dashed lines indicate the performance of the LR model in testing cohort.

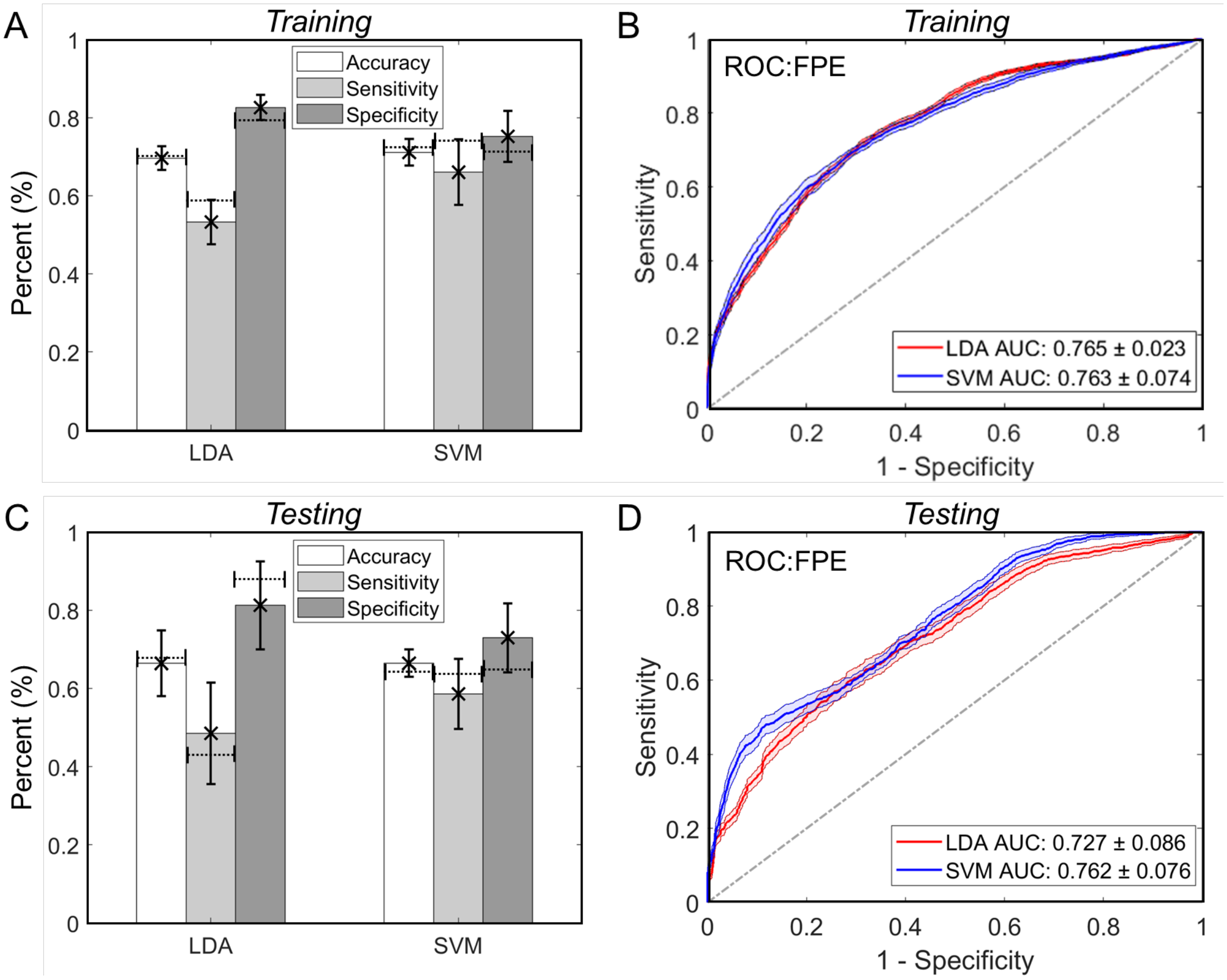

Supplementary Figure S3: LDA (Red) and SVM (Blue) Model Stability Across 100 Randomizations of Training/Testing. (A) Across 100 different randomizations of the selection of the training dataset, the models performed with similar accuracy sensitivity and specificity to their performance in the initial randomization. **(B)** The AUCs measured in the first randomization also fell within the range across the 100 training AUCs. **(C)** In the 100 different testing dataset randomizations the models had an average testing accuracy, sensitivity and specificity similar to their performance in the initial randomization. **(D)** The testing AUCs measured in the first randomization also fell within the range of AUCs across the 100 randomizations.

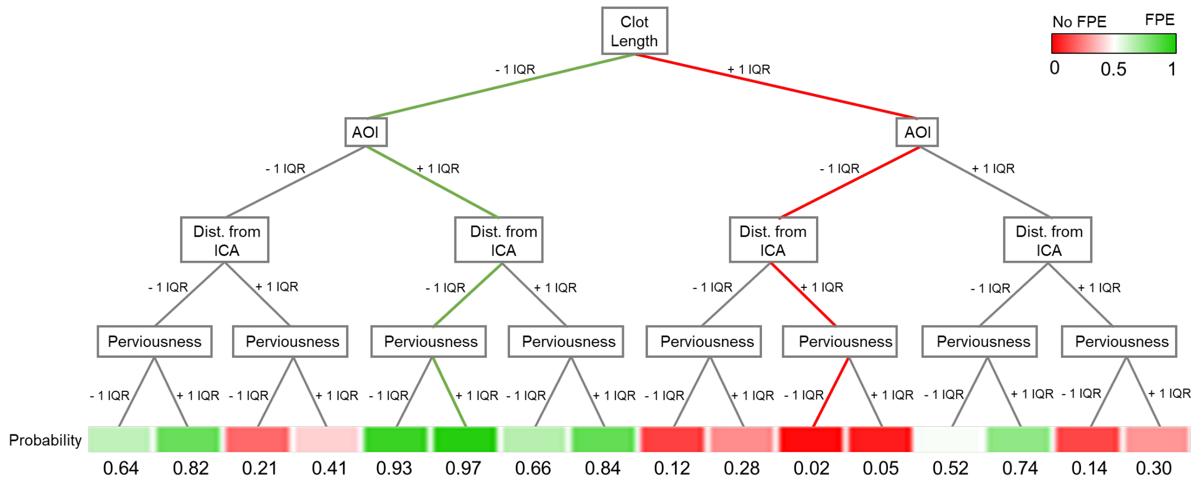

Supplementary Figure S4: Inferred Decision Tree from Trained Logistic Regression Model Coefficients. The decision tree (hierarchy based on parameter importance) depicts the predicted probability with each combination of a 1 IQR increase/decrease in each parameter's median value. In the color map presented at the bottom of the figure, green shading indicates higher probability of a good outcome (FPE), whereas red shading indicates poor outcome probability (no FPE). The green decision branches highlight the highest probability for successful outcome, whereas the red decision branches highlight the lowest probability for successful outcome.
